# Evolution of pathogens towards low *R*_0_ in heterogeneous populations

**DOI:** 10.1016/j.jtbi.2006.04.003

**Published:** 2006-10-07

**Authors:** Rowland R. Kao

**Affiliations:** Department of Zoology, University of Oxford, South Parks Rd., OX1 3PS, UK

**Keywords:** Evolution, Pathogen, Heterogeneous contact, HIV

## Abstract

Maximization of the basic reproduction ratio or *R*_0_ is widely believed to drive the emergence of novel pathogens. The presence of exploitable heterogeneities in a population, such as high variance in the number of potentially infectious contacts, increases *R*_0_ and thus pathogens that can exploit heterogeneities in the contact structure have an advantage over those that do not. However, exploitation of heterogeneities results in a more rapid depletion of the potentially susceptible neighbourhood for an infected host. Here a simple model of pathogen evolution in a heterogeneous environment is developed and placed in the context of HIV transmission. In this model, it is shown that pathogens may evolve towards lower *R_0_*, even if this results in pathogen extinction. For sufficiently high transmissibility, two locally stable strategies exist for an evolving pathogen, one that exploits heterogeneities and results in higher *R_0_*, and one that does not, and results in lower *R_0_*. While the low *R_0_* strategy is never evolutionarily stable, invading strains with higher *R_0_* will also converge to the low *R_0_* strategy if not sufficiently different from the resident strain. Heterogenous transmission is increasingly recognized as fundamental to epidemiological dynamics and the evolution of pathogens; here, it is shown that the ability to exploit heterogeneity is a strategy that can itself evolve.

## 1 Introduction

Theoretical epidemiology is underpinned by the concept of a invasion threshold associated with the basic reproduction ratio or *R*_0_. For *R*_0_>1, a pathogen will be successful, in the sense that the introduction of a single infected individual into a wholly susceptible population will on average result in at least one other infected individual (Anderson and May 1991; Diekmann et al., 1990). Heterogeneities in the available host population that increase the susceptibility and transmissibility (probability of transmission per potentially infectious contact or *τ*) associated with a host subpopulation confer advantages to pathogens that can exploit them, and for a given average transmissibility, results in higher *R*_0_ (Yorke et al., 1978). In the case of sexually transmitted diseases (STD's) for example, highly active and therefore highly connected individuals are both more exposed and cause more infections, and they can play a crucial role in disease spread and persistence (Anderson and May, 1991; Hethcote et al., 1982). In theory, disease can persist even for vanishingly small *τ*, so long as the variance in the number of contacts per individual is sufficiently high (Albert et al. 2000). Thus exploitation of heterogeneities has been suggested as a route by which new pathogens can emerge (May et al. 2001). As the “adaptive model” of evolution is typically driven by maximization of *R*_0_ (Anderson and May, 1982), this also implies that, at least initially, a pathogen able to exploit heterogeneities in the host population will always be favoured over one that does not.

However, a strategy that relies on heterogeneity has disadvantages; the high-risk individuals are infected first, and thus while *R*_0_ is higher, the average number of individuals infected by a single infectious individual at a given time in an epidemic (the reproduction rate *R*(*t*)) declines more quickly if heterogeneities are exploited than if they are not (Barthelemy et al., 2004; Kiss et al., 2006a). This can result in a lower final epidemic size compared to a strategy with the same *τ*, but which does not exploit heterogeneities (Kiss et al., 2006b; May and Lloyd, 2001). Since an evolving pathogen will only be able to exploit *R*(*t*) and not *R*_0_ (Anderson and May, 1982), this suggests that maximization of *R*_0_ does not necessarily predict the direction of evolution. Here a simple, deterministic model of a population with heterogeneous contacts is developed, showing that evolution towards low *R*_0_ may occur, possibly resulting in pathogen extinction even if the starting value of *R*_0_ would predict successful pathogen invasion. In this model, stable states only exist if the pathogen either fully exploits available heterogeneities or does not exploit them at all (i.e. the strain has the same potential to infect all individuals). The final state depends on both the demographic structure and the initial conditions. Implications for epidemic diseases are briefly considered, establishing a plausible mechanism for evolution to low *R*_0_ where disease persistence is maintained by a metapopulation structure. While the high-*R*_0_ strategy is an evolutionarily stable one (ESS), the low-*R*_0_ strategy is not, though invading strains insufficiently differentiated from the prevailing strain are predicted to “converge” towards it.

## 2 Model description—evolution in diseases with *SIRS* or *SIS* dynamics

Heterogeneity in the number of potentially infectious connections is considered, though the model would apply to any form of heterogeneity that equally affects both susceptibility and transmissibility. Consider a population of which a fixed number *N_x_* individuals are highly connected, with the average number of connections scaled by a factor *σ* above the low risk population *N_y_*, individuals of which are poorly connected. The epidemiology is described by a susceptible–infected–removed–susceptible (*SIRS*) system for each of the subpopulations *N_x_* and *N_y_*, with states *, S_x_, S_y_*_,_ *I_x_, I_y_, R_x_*, *R_y_* referring to the states of the subpopulations. Infected individuals either recover and eventually become susceptible again or equivalently are removed and replaced by susceptible individuals in such a way that *N_x_* and *N_y_* are constant. In a population with heterogeneities in the contact structure, each individual in *N_y_* has *κ* potentially infectious contacts or links, and each individual in *N_x_* has *σ*>1 more links than individuals in *N_y_*. Then the expected value of the number of connections per individual over the whole population is

(1) $$\langle \kappa \rangle =\kappa_{\mathrm{av}}=\frac{N_{x}\kappa \sigma +N_{y}\kappa }{N_{x}+N_{y}}\text{.}$$

Assume that the pathogen can exploit this heterogeneity in the contact structure to a variable degree, ranging from full, or a “high-*R*_0_ strategy”, to no exploitation, a “low-*R*_0_ strategy”. Let *z* be the extent to which the low-*R*_0_ strategy is adopted, so that at *z*=1, all individuals potentially infect a number of others given by *κ_av_* in Eq. (1). Therefore *(1-z*) is the proportion of transmission that exploits heterogeneities in the population. Shifts in the net strategy of the pathogen population are governed by the relative proportion of new infections that occur according to each of the two extreme strategies, at a rate determined by a parameter *μ*. The “direction” of evolution is determined by the relative numbers of new infections resulting from each route; in order to retain biologically sensible dynamics, a multiplier of *z*(*1-z*) forces d*z/*d*t* to be zero at *z=*0 and 1. Let the mortality of the infected individuals be *δ* and that of removed individuals be *ε* and recall that the probability of transmission per connection is τ. Then the model system can be described by a set of differential equations:

$$\frac{dI_{x}}{dt}=F_{1}\left(I_{x},I_{y}\right)+F_{2}\left(I_{x},I_{y}\right)-\delta I_{x}\text{,}\frac{dI_{y}}{dt}=G_{1}\left(I_{x},I_{y}\right)+G_{2}\left(I_{x},I_{y}\right)-\delta I_{y}\text{,}\frac{dR_{x}}{dt}=\delta I_{x}-\varepsilon R_{x}\text{,}\frac{dR_{y}}{dt}=\delta I_{y}-\varepsilon R_{y}\text{,}\frac{dz}{dt}=\mu \tau z\left(1-z\right)(F_{2}\left(I_{x},I_{y}\right)+G_{2}\left(I_{x},I_{y}\right)+F_{1}\left(I_{x},I_{y}\right)-G_{1}\left(I_{x},I_{y}\right))\text{,}F_{1}\left(I_{x},I_{y}\right)=\left(1-z\right)\tau \left(\kappa \sigma I_{x}+\kappa I_{y}\right)\frac{\kappa \sigma \left(N_{x}-I_{x}\right)}{N_{x}\kappa \sigma +N_{y}\kappa }\text{,}F_{2}\left(I_{x},I_{y}\right)=z\tau \kappa_{\mathrm{av}}\left((I_{x}+I_{y}\right)\left(\frac{N_{x}-I_{x}}{N}\right)\text{,}G_{1}\left(I_{x},I_{y}\right)=\left(1-z\right)\tau \left(\kappa \sigma I_{x}+\kappa I_{y}\right)\frac{\kappa \left(N_{y}-I_{y}\right)}{N_{x}\kappa \sigma +N_{y}\kappa }\text{,}G_{2}\left(I_{x},I_{y}\right)=z\tau \kappa_{\mathrm{av}}\left(I_{x}+I_{y}\right)\left(\frac{N_{y}-I_{y}}{N}\right)\text{,}N_{j}=S_{j}+I_{j}j=x,y(\mathrm{const})\text{,}0⩽z⩽1\text{,}\sigma >1\text{.}$$

Taking proportions of populations *x=I_x_/N_x_*, *y=I_y_/N_y_*, *r_x_=R_x_/N_x_, r_y_=R_y_/N_y_, f=N_x_/N,* letting $\tau \kappa_{y}/\delta =\gamma$ and η=ε/δ and assuming dimensionless time (equivalently, setting the removal rate of infected individuals δ=1), the model can be expressed as

(2a) $$\frac{dx}{dt}=\gamma (\left(1-z\right)\left(\sigma x+y\right)\frac{\sigma \left(f-x-r_{x}\right)}{f\sigma +1-f}+z\left(f\sigma +1-f\right)\left(x+y\right)\left(f-x-r_{x}\right))-x$$

(2b) $$\frac{dr_{x}}{dt}=x-\eta r_{x}\text{,}$$

(2c) $$\frac{dy}{dt}=\gamma (\left(1-z\right)\left(\sigma x+y\right)\frac{1-f-y-r_{y}}{f\sigma +1-f}+z\left(f\sigma +1-f\right)\left(x+y\right)\left(1-f-y-r_{y}\right))-y\text{,}$$

(2d) $$\frac{dr_{y}}{dt}=y-\eta r_{y}\text{,}$$

(2e) $$\frac{\mathrm{dz}}{\mathrm{dt}}=\mu \gamma z\left(1-z\right)(z\left(f\sigma +1-f\right)\left(x+y\right)\left(1-x-y-r_{x}-r_{y}\right)-\left(1-z\right)\left(\sigma x+y\right)\left(\frac{\left(f-x-r_{x}\right)\sigma }{f\sigma +1-f}+\frac{1-f-y-r_{y}}{f\sigma +1-f}\right))\text{.}$$

If there is no recovery/removed stage (SIS model), this becomes a system of three equations:

(3a) $$\frac{dx}{dt}=\gamma (\left(1-z\right)\left(\sigma x+y\right)\frac{\sigma \left(f-x\right)}{f\sigma +1-f}+z\left(f\sigma +1-f\right)\left(x+y\right)\left(f-x\right))-x\text{,}$$

(3b) $$\frac{dy}{dt}=\gamma (\left(1-z\right)\left(\sigma x+y\right)\frac{1-f-y}{f\sigma +1-f}+z\left(f\sigma +1-f\right)\left(x+y\right)\left(1-f-y\right))-y\text{,}$$

(3c) $$\frac{dz}{dt}=\mu \gamma z\left(1-z\right)(z\left(f\sigma +1-f\right)\left(x+y\right)\left(1-x-y\right)-\left(1-z\right)\left(\sigma x+y\right)\left(\frac{\left(f-x\right)\sigma }{f\sigma +1-f}+\frac{1-f-y}{f\sigma +1-f}\right))\text{.}$$

As the main interest is in evolution of the pathogen, and the stability results for *z* are unchanged between the *SIRS* and *SIS* models since d*z/*d*t* does not depend on the removed population, the remainder of the analysis will concentrate on the three equation *SIS* system. Finally, a division of populations is chosen such that for all scalings, the fraction of highly connected individuals is fixed so that the average susceptibility is fixed as twice the low risk susceptibility, i.e. f=1/(σ-1) with σ⩾2.

## 3 Competition between strains and evolutionarily stable strategies

Thus far, the model presented only considers the convergence stability of a single evolving strain, or the behaviour of a “cloud” of strains none of which diverge significantly from the mean strategy. In this case, the equations are rewritten to allow a second strain with complete cross-immunity between the two strains, and indicated by primes:

$$\frac{dx}{dt}=\gamma (\left(1-z\right)\left(\sigma x+y\right)\frac{\sigma \left(f-x-x^{\prime }\right)}{f\sigma +1-f}+z\left(f\sigma +1-f\right)\left(x+y\right)\left(f-x-x^{\prime }\right))-x\text{,}\frac{dy}{dt}=\gamma (\left(1-z\right)\left(\sigma x+y\right)\frac{1-f-y-y^{\prime }}{f\sigma +1-f}+z\left(f\sigma +1-f\right)\left(x+y\right)\left(1-f-y-y^{\prime }\right))-y\text{,}\frac{dx^{\prime }}{dt}=\gamma (\left(1-z^{\prime }\right)\left(\sigma x^{\prime }+y^{\prime }\right)\frac{\sigma \left(f-x-x^{\prime }\right)}{f\sigma +1-f}+z\left(f\sigma +1-f\right)\left(x^{\prime }+y^{\prime }\right)\left(f-x-x^{\prime }\right))-x^{\prime }\text{,}\frac{dy^{\prime }}{dt}=\gamma (\left(1-z\right)\left(\sigma x^{\prime }+y^{\prime }\right)\frac{1-f-y-y^{\prime }}{f\sigma +1-f}z+z^{\prime }\left(f\sigma +1-f\right)\left(x^{\prime }+y^{\prime }\right)\left(1-f-y-y^{\prime }\right))-y^{\prime }\text{,}\frac{dz}{dt}=\mu \gamma z\left(1-z\right)(z\left(f\sigma +1-f\right)\left(x+y\right)\left(1-x-x^{\prime }-y-y^{\prime }\right)-\left(1-z\right)\left(\sigma x+y\right)\left(\frac{\left(f-x-x^{\prime }\right)\sigma }{f\sigma +1-f}+\frac{1-f-y-y^{\prime }}{f\sigma +1-f}\right))\text{,}\frac{dz^{\prime }}{dt}=\mu \gamma z^{\prime }\left(1-z^{\prime }\right)(z^{\prime }\left(f\sigma +1-f\right)\left(x^{\prime }+y^{\prime }\right)\left(1-x-x^{\prime }-y-y^{\prime }\right)-\left(1-z^{\prime }\right)\left(\sigma x^{\prime }+y^{\prime }\right)\left(\frac{\left(f-x-x'\right)\sigma }{f\sigma +1-f}+\frac{1-f-y-y^{\prime }}{f\sigma +1-f}\right))\text{.}$$

In order to consider competition between strains, it is necessary to evaluate the basic reproduction number $R_{0}(z^{\prime },s^{*})$ of a new invading strain, in the presence of a prior existing strain, where *z′* is the extent to which the new strain exploits heterogeneity in the host population, and $s^{*}=1-x^{*}-y^{*}$ is the susceptible population when the initial strain is at equilibrium. If $R_{0}(z^{\prime },s^{*})<1$ for all *z′*, then the initial strain is an ESS and is resistant to invasion by any competing strain within the context of the model. Note that for the single strain case, $R_{0}=R_{0}(z^{\prime },1)$.

## 4 Results

### 4.1 Equilibria and local convergence stability

The mutation rate *μ* is only a multiplicative factor for Eqs. (2e) and (3c), and thus does not affect the existence of local stability, though it can affect the size of the locally stable regions. Eq. (3c) is of the form dz/dt=z(1-z)a(z)*,* where *a*(*z*) is a linear increasing function of *z*. Thus for any given coordinates *(x_0_, y_0_)*, there are at most three fixed points in *z*: at the extrema z=0*,* and 1 and at one intermediate point, (*x*_0_*, y*_0_*, z*_0_) where the set of all these points is defined by the surface a(z)=0. As *a*(*z*) is a linear function, the intermediate fixed point must be unstable as for *z* values below the surface defined by a(z)=0, the value of *a*(*z*) and therefore d*z/*d*t* is strictly negative, and above the surface they are strictly positive. Since this is true for all (*x_0_, y_0_*), there are no intermediate stable fixed points for a given value of *z*.

The remaining two fixed points are identified by the solution of the system at z=0:

$$y=\frac{\sigma x^{2}-\left(\phi \sigma^{2}f-1/\phi \sigma \right)x}{\left(f-x\right)}\text{,}x=\frac{\left(\phi \left(1-f\right)-1\right)y-\phi y^{2}}{\left(\phi \sigma \right)y-\phi \sigma \left(1-f\right)}\text{,}\phi =\frac{\gamma }{f\sigma +1-f}$$

and at z=1:

$$x+y=1-\frac{1}{\gamma \left(f\sigma +1-f\right)}\text{,}\frac{x}{f}=\frac{y}{1-f}\text{.}$$

**Fig. 1:**
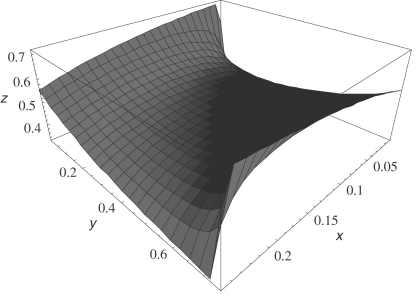
The dz/dt=0 surface. Above the surface, the direction of evolution is upwards, while below the surface, the direction of evolution is downwards. Axes are the proportion of the total population that is high risk and infected (*x*), the proportion of the population that is low risk and infected (*y*), and the extent to which the transmission dynamics affects all individuals equally (z=0 indicating full exploitation of population heterogeneities, and z=1 indicating no exploitation of heterogeneities and all individuals equally likely to become infected). The surface is a saddle, with maxima and minima at the edges of the surface. The scaling factor (increased risk of the subpopulation x) is σ=5 and the proportion of population that is high risk is f=1/4.

Fig. 1 shows the surface defining dz/dt=*0* (excluding z=0 and 1). A transformation v=sx+y*,* and w=x+y, shows that this surface is defined by

(4) $$z_{0}\left(v,w\right)=\frac{(2-v)v}{4(1-w)w+(2-v)v}\text{.}$$

In general, the trajectory of a solution in *(x, y, z)* space is not restricted to fixed values of *z.* A trajectory may “pierce” the surface defined by Eq. (4) (potentially more than once) and so the initial direction of evolution does not necessarily predict the final state. Evaluation of the gradient $\nabla (z_{0}(v,w)),$ shows this surface has a single fixed point at $\left(v_{0},\omega_{0})=(1,\frac{1}{2}\right)$. The determinant of the Hessian Matrix

$$H\left(z_{0}\right)=\left[\begin{array}{cc} \frac{\partial^{2}z_{0}}{\partial v^{2}} & \frac{\partial^{2}z_{0}}{\partial v\partial w}\\ \frac{\partial^{2}z_{0}}{\partial w\partial v} & \frac{\partial^{2}z_{0}}{\partial w^{2}} \end{array}\right]$$

at (*v_0_, w_0_*) is strictly negative, and by definition the fixed point is therefore a saddle point (e.g. Barr, 2000). For $R_{0}(z,1)>1$, there is therefore a region of local stability for both the purely low-*R*_0_ strategy and high-*R*_0_ strategies, provided z>Max(z|dz/dt=0) and z<Min(z|dz/dt=0) respectively, which are defined at the boundaries of the *x−y* solution space (i.e. combinations of x=0 or x=f, and y=0 or y=1-f).

### 4.2 The basic reproduction number

The basic reproduction number or *R*_0_ is typically defined in the epidemiology literature as the number of secondary cases resulting from a single infected primary case, introduced into a wholly susceptible population at equilibrium (Anderson and May, 1991). Using the next generation matrix definition (Diekmann et al., 1990), it is more generally the ratio of the number of individuals in all infected classes in successive generations, in the limit of a large number of generations and a large population, i.e.: $R_{0}=\lim_{N,n\to \infty }\left(I_{n+1}/I_{n}\right),$ where *N* is the population size, *n* is the generation number, and *I_n_* is the number of infected individuals in all classes in generation *n*.

If a single node is infected at random, then the average number of secondary cases created by this index case is $\rho_{0}=\sum_{\kappa }\tau \kappa p\left(\kappa \right)$ where the summation is over all possible values of the number of connections *κ*. To calculate *R*_0_, let the number of infected nodes of degree *i* in generation *j* be *I_i,j_* . If the links amongst individuals are fixed, then the degree of the infected node should be reduced due to the link to the original infecting node which now cannot infect anyone. Should turnover of links be fast relative to the incubation period and/or infectious period, then this effect will be reduced, ultimately to zero; see also (Dietz and Hadeler, 1988). For example, if the links represent sexual partnerships, the incubation period is 1 month, and (unrealistically) all partnerships last less than 1 month, then there will be minimal overlap in the pattern of partnerships when the individual is infected, as compared to when the individual is infectious, assuming that links are formed randomly, and this is the assumption made here. Then in the subsequent generation, $I_{\kappa_{j+1},j+1}=\sum_{\kappa_{j}}\tau \kappa_{j}P\left(\kappa_{j+1}|\kappa_{j}\right)I_{\kappa_{j},j},$ where $P(\kappa_{j+1}|\kappa_{j})$ is the probability that a link from a node of degree *κ_j+1_* is connected to a node of degree *κ_j_*. As the network here is randomly connected, in the first generation $P\left(\kappa_{1}|\kappa_{0}\right)=\kappa_{1}p\left(\kappa_{1}\right)/\langle \kappa \rangle ,$ i.e. it does not depend on *κ*_0_. The number of infected elements of an arbitrary degree *κ*_1_ in the first generation of transmission is then:

$$I_{\kappa_{1},1}=\frac{\tau \kappa_{1}p\left(\kappa_{1}\right)\sum_{k_{0}}\kappa_{0}p\left(\kappa_{0}\right)}{\langle \kappa \rangle }=\tau \kappa_{1}p\left(\kappa_{1}\right)$$

and in the following generation is

$$I_{\kappa_{2},2}=\underset{k_{1}}{\sum }\tau \left(\kappa_{1}-1\right)I_{\kappa_{1},1}p\left(\kappa_{2}|\kappa_{1}\right)=\tau^{2}\kappa_{2}p\left(\kappa_{2}\right)\frac{\langle \kappa^{2}\rangle }{\langle \kappa \rangle }\text{.}$$

Summing over all connections in both cases, $I_{1}=\tau \sum_{\kappa_{1}}\kappa_{1}p\left(\kappa_{1}\right)=\tau \langle \kappa \rangle$ and similarly $I_{2}=\tau^{2}\langle \kappa^{2}\rangle$. It is easy to show that the ratio $I_{2}/I_{1}=I_{n+1}/I_{n}$ for all subsequent successive generations *n* and n+1, and therefore $R_{0}=\tau \langle \kappa^{2}\rangle /\langle \kappa \rangle$ (equivalent to the result for sexually transmitted diseases and other expressions derived for networks, see for example, Diekmann and Heesterbeek (2000)). Under the assumed form here,

$$\langle \kappa^{2}\rangle =f\kappa^{2}\sigma^{2}+\left(1-f\right)\kappa^{2}=f{\left(\left(1-z\right)\kappa \sigma +z\left(f\kappa \sigma +\left(1-f\right)\kappa \right)\right)}^{2}+\left(1-f\right)(\left(1-z\right)\kappa +{z\left(f\kappa \sigma +\left(1-f\right)\kappa \right))}^{2}\text{,}\langle \kappa \rangle =f\kappa \sigma +\left(1-f\right)\kappa =f\left(\left(1-z\right)\kappa \sigma \right)+\left(1-f\right)\left(\left(1-z\right)\kappa \right)+z\left(f\kappa \sigma +\left(1-f\right)\kappa \right)$$

Therefore using the scaling restriction f=1/(σ-1), then $R_{0}=R_{0}$ (z, 1) at a fixed value of *z* is

$$R_{0}\left(z,1\right)=\gamma \left(z^{2}\left(\frac{\sigma -2}{2}\right)+z\left(2-\sigma \right)+\left(\frac{\sigma +2}{2}\right)\right)\text{.}0<z<1\text{,}\sigma >2\text{,}$$

which is a maximum at z=0 and monotonically decreasing in *z*. Thus the existence of a locally stable steady state (*x_s_, y_s_, z_s_=*1) implies the existence of a region where pathogens can successfully evolve to lower *R*_0_. Under conditions of pathogen introduction when there is a prevailing endemic strain, not only the proportion of susceptibles (s^*^) must be considered, but also the relative proportions of highly connected (s^*^_x_) and poorly connected (s^*^_y_) individuals must be considered. In this case,

(5a) $$R_{0}\left(z,s^{*}\right)=\gamma \frac{{\left(\left(1-z\right)\sigma \frac{s_{x}^{*}\sigma +s_{y}^{*}}{2}+z\kappa_{\mathrm{av}}s^{*}\right)}^{2}s_{x}^{*}+{\left(\left(1-z\right)\frac{s_{x}^{*}\sigma +s_{y}^{*}}{2}+z\kappa_{\mathrm{av}}s^{*}\right)}^{2}s_{y}^{*}}{\left(\left(1-z\right)\sigma \frac{s_{x}^{*}\sigma +s_{y}^{*}}{2}+z\kappa_{\mathrm{av}}s^{*}\right)s_{x}^{*}+\left(\left(1-z\right)\frac{s_{x}^{*}\sigma +s_{y}^{*}}{2}+z\kappa_{\mathrm{av}}s^{*}\right)s_{y}^{*}}\text{,}$$

(5b) $$s_{x}^{*}+s_{y}^{*}=s^{*}\leq 1\text{.}$$

### 4.3 Convergence stability and evolution in a metapopulation model with SIR dynamics

Evolution of pathogens in epidemic diseases can differ from that for endemic diseases. For the *SIR* epidemic model there is by definition no endemic equilibrium, as there is no recovery of infected individuals to maintain the population of susceptibles. Therefore there is no unique final evolutionary state, if only a single population is considered. Persistence can be maintained in a metapopulation model, if it is assumed that a population patch will be repopulated by susceptible hosts after disease extinction has occurred. Individual pathogen population extinctions are balanced by pathogen migration which occurs at a different time-scale, allowing exhausted patches to be repopulated with susceptible hosts—in this sense it is more correctly an *SIRS* model with very slow host repopulation rates (Levins and Culver, 1971). While it is beyond the scope of this paper to explore the full consequences of the metapopulation model, it is clear that Eq. (3c) has the same dependencies whether susceptible renewal is allowed, or not. Therefore, there are no intermediate stable fixed points for *z*, and there are local regions bounded below by z=0 where the evolution of *z* is strictly negative whatever the initial values of *x* and *y*, with a similar region bounded above by z=1, where the evolution of *z* is strictly positive. Thus, should the pathogen evolve to a state that lies in the stable regions for all subpopulations, the pathogen in the metapopulation model will eventually evolve to either z=0 or 1, provided it does not go extinct.

### 4.4 Evolutionary Stability

**Fig. 2:**
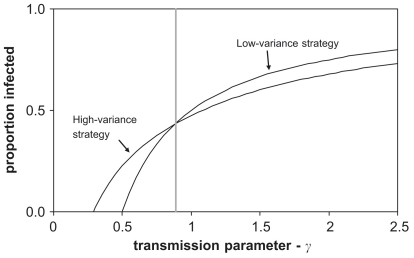
Proportion of population infected as a function of the normalised transmission rate. The high-variance strategy (exploiting heterogeneities in the population) persists at lower transmission rates and initially infects a greater proportion of the population, but as the transmission rate increases is at a disadvantage compared to the low-variance strategy.

**Fig. 3:**
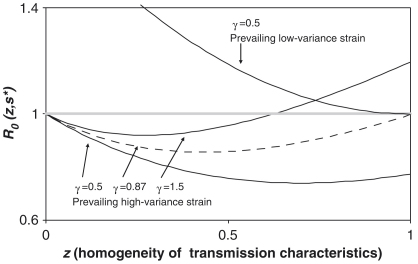
Value of the basic reproduction ratio (*R*_0_(*z*, s*) under two different prevailing strains and for varying *z* (exploitation of heterogeneity). The prevailing strain infects a proportion (1-s*) of the population. For sufficiently high transmission rate g, a prevailing low-variance strain can always be invaded by a strain that exploits heterogeneity (i.e. *R*_0_(*z*, s*)>1 for z<1). The high-variance strain can be invaded by a low-variance strain for high *z*, dependent on the value of *γ*.

Fig. 2 shows the increase in the equilibrium prevalence when z=0 and 1, as *γ* increases. Mirroring the result for the final epidemic size in the SIR model (May and Lloyd, 2001), above a critical value *γ_crit_*, the low-*R*_0_ strategy has a higher endemic equilibrium than the high-*R*_0_ strategy, as the latter “uses up” the highly connected individuals more efficiently. Intuitively, this implies that above *γ_crit_*, a low-*R*_0_ strain should be able to invade a prevailing, endemic, high-*R*_0_ strain. This is confirmed using Eq. (5); Fig. 3 shows that, for sufficiently low *γ* , the high-*R*_0_ strategy is an ESS, but above *γ_crit_* strains exploiting lower *R*_0_ strategies are able to invade. In contrast, when the prevailing strain is low-*R*_0_, strains that exploit heterogeneity are always able to invade. Intriguingly however, such invading strains may evolve towards a low-*R*_0_ strategy (effectively, they are assimilated into the prevailing population).

### 4.5 Novel introduction into a wholly susceptible population

Specific stability results are dependent on the initial conditions, however consideration of a particular example provides some insight; in this case, the novel introduction of the pathogen into a wholly susceptible population. It is assumed that the pathogen has previously evolved in a population with differing demographic characteristics, so that effectively it is at an intermediate value of *z*. The example considers introduction of a single infected individual belonging to subpopulation *x* into a population of 10,000 with scaling σ=5 and mutation rate μ=1.

**Fig. 4:**
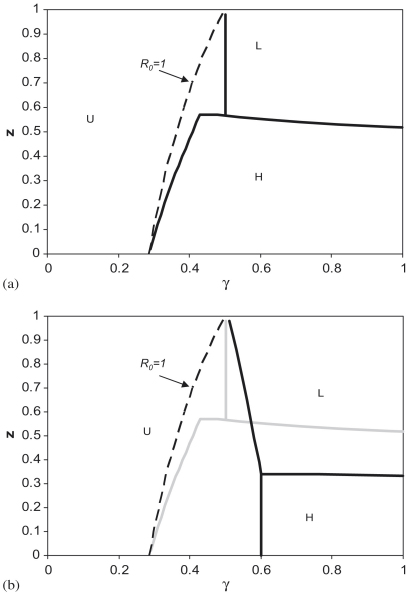
Phase representation of the final state following introduction of disease in a single, highly susceptible individual. The phase diagram shows regions in which there is no stable steady state (U), where a high variance strategy is stable (H), and where a low variance strategy is stable (L). Without control (a), one quarter of the population is five times as susceptible as the rest, with targeted disease control in place (b), one quarter of the population is only 3 times as susceptible. In figure 4b, the original phase diagram without control is shown in grey for comparison. The shift in relative susceptibility can make pathogen evolution to the “low variance strategy” more likely. The dashed line indicates $R_{0}=1$.

**Fig. 5:**
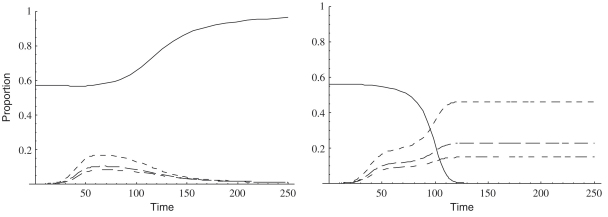
Time evolution of disease following introduction of a single highly connected individual into a population of 10 000, showing evolution to extinction. On the left, the pathogen evolves towards a low-variance strategy (starting value z=0.57, solid line) and drives itself to extinction, on the right the pathogen evolves towards the high-variance strategy and persists (z=0.56). The remaining curves show the proportion of highly connected (high risk) population infected (short dashes), proportion of poorly connected (low risk) population infected (dot–dash), and proportion of total population infected (long dashes). Parameters are scaling factor σ=5, and infection rate γ=0.5 and population shift rate μ=1.0.

Fig. 4 shows the resultant phase diagram in *z* and *γ* under these parameters (Fig. 4a), and with the same parameters but with the scaling factor reduced to σ=3 (Fig. 4b). This is equivalent to changing the behaviour of a targeted proportion of the population (e.g. increased use of prophylactics in the high risk population to prevent STD transmission). For $R_{0}(z,1)<1$, the disease-free equilibrium is the only steady-state. As *γ* increases, the high variance strategy (z=0) becomes locally stable, but the low-*R*_0_ strategy (z=1) remains unstable. The locally stable region around z=1 appears as soon as the low-*R*_0_ strategy becomes viable (i.e. $R_{0}(z,\;1){|}_{z=1}>1$). Fig. 4 shows the existence of a region where $R_{0}(z,1)>1$, but the solution can still tend towards the z=1, $R_{0}(z,1)<1$ regime, and thus the pathogen disappears. This is illustrated in Fig. 5. At higher *γ* the low-*R*_0_ strategy becomes more likely, but dependence on *γ* is not strong (less than a 5% increase in the locally stable region, for a doubling of *γ* from 0.5 to 1). Changing the scaling factor *s* (i.e. decreasing the extent of heterogeneity) can have a more dramatic effect on the phase diagram, as reducing *σ* from 5 to 3 increasing the stable regime by approximately 20% at γ=1.0.

**Fig. 6:**
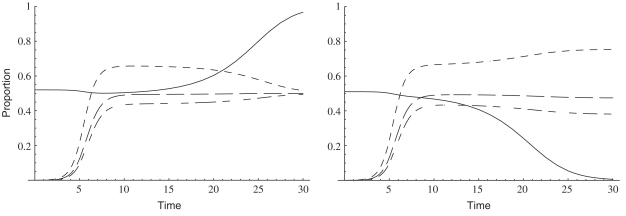
Time evolution of disease following introduction of a single highly connected individual into a population of 10 000. The curves show evolution of heterogeneity (solid), proportion of highly connected (high risk) population infected (short dashes), proportion of poorly connected (low risk) population infected (dot–dash), and proportion of total population infected (long dashes). Parameters are scaling factor σ=5, and infection rate γ=1.0 and population shift rate μ=1.0. The two solutions differ only in the starting value of the heterogeneity variable *z*, starting at $z_{0}=0.52$ on the left, and $z_{0}=0.51$ on the right.

An examination of the time course of the epidemic where both strategies can be successful (Fig. 6) shows that highly connected individuals become infected more rapidly, but as the pathogen evolves towards the low-*R*_0_ strategy the proportion of highly infected can decrease, though the overall proportion of the population infected will be higher, similar to previous results for investigations in populations with scale-free distributions of connections (Barthelemy et al., 2004; Kiss et al., 2006a). As expected, a slight shift in the initial conditions can change the direction of evolution. Note that the initial direction of evolution does not predict the final state.

## 5 Discussion

The restriction to evolution of pathogens with fixed transmissibility per act is a device to explore the consequences of allowing pathogen exploitation of host heterogeneity to evolve, and is not a real restriction on possible directions of pathogen evolution. It is used here to illustrate how the relationship between demographics and transmission characteristics is vital to understanding disease evolution—transmission characteristics alone are insufficient, as, in the absence of disease control, any pattern of potentially infectious contacts (the “social network”) plus transmission probabilities per contact that generates the same pattern of truly infectious contacts (the “epidemiological network”) will have the same characteristics (Kao et al., 2006). It has been suggested (Frank, 1996) that endemic diseases favour prolonged infectious periods (in this case, more homogeneous transmission characteristics) at the expense of viral replicative fitness (more heterogeneous transmission characteristics), while epidemic diseases favour the converse. However, here it has been shown that regions of local stability exist for both strategies that depend only on the demographic parameters, and not on the disease prevalence or whether the disease is endemic (*SIS*) or epidemic (*SIR*), implying that both strategies are potentially viable. Taking into account exploitable heterogeneities in the population, epidemic diseases can potentially evolve to lower viral replication rates and longer infectious periods, and endemic diseases the converse. Real pathogens, of course, will have limitations on their evolution not considered in this model (Brander and Walker, 2003), and the epidemiological consequences must be viewed in the context of both within-host and between-host considerations.

While the model is expressed in general terms of heterogeneity, the example of HIV transmission provides a plausible application. It is well known that the distribution of human sexual contacts has high variance (Liljeros et al., 2001). Though the exact form of the distribution is the subject of some dispute (Jones and Handcock, 2003), this distribution has been posited as the driving force behind the emergence and spread of HIV (May et al., 2001). The number of partners and duration of partnership appear to be negatively correlated (Ghani et al., 1997; Kretzschmar, 2000). While repeated exposure to infected partners would be expected to increase the risk of becoming infected, viral loads, believed to be a good indicator of infectiousness (Pedraza et al., 1999), decline quickly and dramatically, and infectiousness per heterosexual coital act has been estimated to drop as much as an order of magnitude within six months post-infection (Wawer et al., 2005), though there is a rise in infectiousness as the infected individual approaches the terminal stages of disease. Thus the effect of multiple exposures in long term partnerships is mitigated, and the number of partnerships and not number of acts remains the key epidemiological parameter (Anderson and May, 1988). There is recent evidence however, that HIV-1 may be evolving towards lower viral replicative fitness (Arien et al., 2005), suggesting decreased pathogenicity of HIV-1 over time. However, if lower pathogenicity (presumably resulting in a lower probability of transmission per act) is accompanied by a longer infectious period, individuals involved in relatively few longer term partnerships with greater exposure would have an increased risk of infection per partnership than individuals involved in many short term partnerships. Thus while the social network pattern is unchanged, the changes in the transmission characteristics result in a different epidemiological network, equivalent here to increasing *z*.

For sufficiently low values of *τ* the system can evolve from one of successful disease invasion $\left(R_{0}>1\right)$ to one where the disease cannot successfully invade $\left(R_{0}<1\right)$, as the solution drifts towards more homogeneous transmission. The prediction that there are no stable mixed strategies reflects an earlier result which show that mixed strategies result in lower final epidemic size than either wholly high- or low-*R*_0_ strategy (Kiss et al., 2006b). Of course, the underlying mechanism here only captures the characteristics of pathogen evolution in an abstract fashion. More detailed approaches such as via individual-based models and the addition of stochastic effects will undoubtedly provide further illumination.

While exploitation of heterogeneity is the only successful strategy for low *τ*, as *τ* increases, evolution towards a stable low-*R*_0_ strategy becomes possible. While this strategy is not an ESS, it is robust in the sense that there is local convergence towards the strategy. Evolution of pathogen virulence in the presence of imperfect vaccination has previously been discussed (Gandon et al., 2001). Here it is suggested that changes in the demographic structure of a population, whether by behavioural changes, invasion of a new population or through imperfect, targeted disease control could drive a pathogen towards lower *R*_0_, but result in a larger proportion of the population becoming infected. This difference is driven by the variable rate of exploitation of the different subpopulations, with depletion of high risk susceptibles changing the characteristics of the available, susceptible neighbourhood. The role of *R_0_* maximisation is often emphasised in our understanding of pathogen fitness, and this is sensible in establishing the minimum pathogen invasion threshold. However, pathogens rarely evolve in a wholly susceptible or unchanging environment; the “local neighbourhood” may be significantly different from the wholly susceptible one that lies behind the *R*_0_-based hypothesis, resulting in a more complicated picture of the mechanisms behind evolutionary dynamics.
